# A well-hidden esophageal foreign body in an 11-year-old boy treated for gastroesophageal reflux disease: a case report

**DOI:** 10.11604/pamj.2022.42.165.34154

**Published:** 2022-06-30

**Authors:** Dimitrios Godosis, Maria Fotoulaki, Chrysostomos Kepertis, Ioannis Spyridakis

**Affiliations:** 1 2^nd^ Department of Paediatrics Surgery, Aristotle University of Thessaloniki, “Papageorgiou” General Hospital, Thessaloniki, Greece,; 2 4^th^ Department of Paediatrics, Aristotle University of Thessaloniki, “Papageorgiou” General Hospital, Thessaloniki, Greece

**Keywords:** Foreign body, ingestion, esophageal obstruction, children, case report

## Abstract

Symptoms of gastroesophageal reflux disease (GERD) in children with developmental disorders could be often confusing. Especially when considering accidental foreign body ingestion, with no acute signs or symptoms of choking. We hereby present a case of an 11-year-old male with a well-hidden esophageal foreign body who was already treated for GERD and finally diagnosed with a hidden foreign esophageal object, ingested 12 months ago. Ingestion of objects of any kind, especially in certain groups of children, must always raise high suspicion for the clinical pediatric specialties, regardless of the presence or absence of classic symptoms.

## Introduction

Gastroesophageal reflux disease (GERD) represents a common pathology among children. However, signs and symptoms may confuse the pediatrician, as there may conceal life-threatening entities. An esophageal foreign object, accidentally ingested by an 11-year-old boy, is presented in the following case.

## Patient and observation

**Patient information:** an 11-year-old male was admitted to our pediatric gastroenterology department for evaluation of dysphagia. Food impaction had occurred 10 days ago and is still ongoing. The child was fed only with liquids. Nausea with vomiting and food refusal was present for the past 12 months, and the patient was treated unsuccessfully with various medications for GERD. According to his personal history, he was a boy with significant learning and behavioral problems, mainly hyperactivity.

**Clinical findings:** physical examination, as well as biochemical and hematological exams, revealed no pathology.

**Figure 1:**
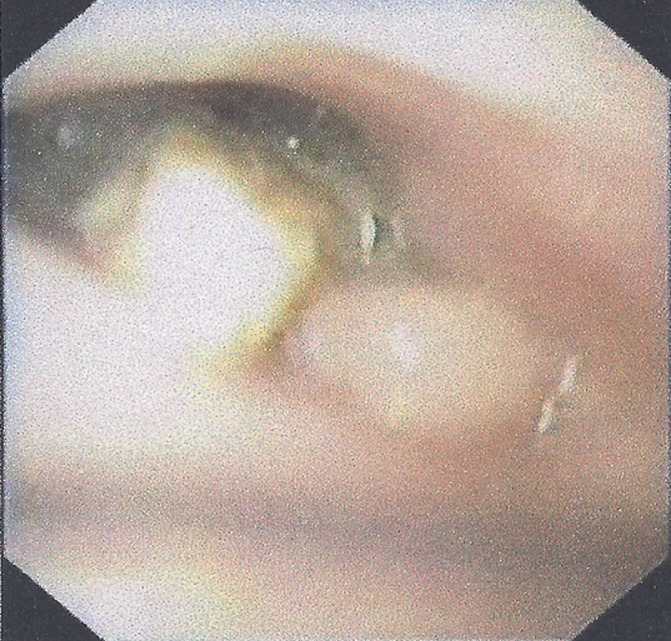
10 cm distally from the upper esophageal sphincter, a rigid polypoid body was discovered, with a slightly inflamed base and subsequent difficulty of passing the endoscope further

**Diagnostic assessment:** chest X-ray (anteroposterior and lateral) revealed no abnormal findings. Endoscopy of the upper gastrointestinal tract showed a scar tissue formation in the middle of the esophagus with a pointed core at the center. Figure 1 attempts to retrieve the core out of this lesion both with flexible and rigid endoscopes failed. Recent medical history was negative for esophageal injury or other pathology. At that stage of the workup, the parents reported that their son had accidentally bitten a piece of glass while drinking, 12 months ago. No acute signs related to glass ingestion had been noted, however retrospectively parents had the idea that nausea and vomiting had insidiously developed since then.

**Diagnosis:** following the above information, we suspected that the endoscopic findings represented a piece of glass with healing scar tissue around it. Immediate surgical exploration was proposed and conducted.

**Figure 2:**
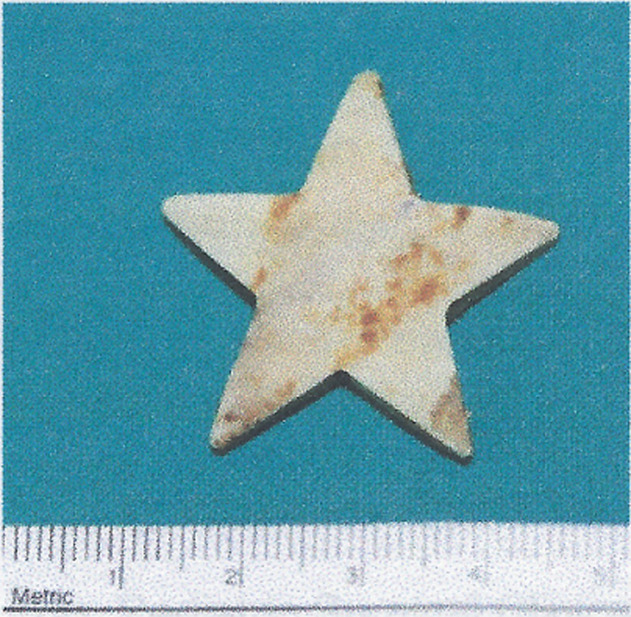
the plastic, star-shaped ingested object, finally retrieved after surgical removal

**Therapeutic intervention:** the esophagus was approached through a right thoracotomy and longitudinal incision to the upper third of the esophagus. However, the foreign body was not a piece of glass, but a star-shaped, flat flexible plastic toy. Figure 2 the immediate post-operative period was uneventful.

**Follow-up and outcomes:** a follow-up esophagoscopy four weeks later had normal findings and since then the child remained asymptomatic.

**Patient's perspective:** the patient himself was anxious. His sentimental status was exaggerated, especially due to his neurodevelopmental disorder. Fortunately, his mother's presence played an important role in comforting him during his hospitalization.

**Informed consent:** the patient's mother was informed about the case report and the reasons why this incident was interesting for the authors. She gave informed consent, allowing the authors to use the boy's story and picture.

## Discussion

Foreign body ingestion is a common complaint in the pediatric emergency department in toddlers and teenagers, especially those with emotional disturbances or mental retardation. Ingestion is under reported or unwitnessed in 40% of cases. Most incidents do not have consequences, as the objects pass spontaneously, while 10%-20% of cases require some intervention, and only 1% demand a surgical approach [1]. Presenting symptoms in children with ingested esophageal objects include vomiting, blood in saliva, neck pain, dysphagia, food refusal, coughing, or strider. If an object remains lodged in the esophagus for more than 2 weeks, there is a significant risk of erosion into surrounding tissues and other serious adverse effects [1,2]. However, rare cases with neglected objects in the esophagus, that remained asymptomatic for years, have been reported [3]. Reilly *et al*.report that, among the population of children with neurodevelopmental disorder, the higher risk of ingestion is related to reasons such as prolonged oral phase, dysphagia with limited control over objects put in the oral cavity, communication impairment, or poor control of hand-to-mouth activity [4,5]. Medical history, symptoms, and a plain X-ray usually establish the diagnosis. In the case of radiolucent objects, that cannot be detected in a plain X-ray, endoscopy has a major role in diagnosis and treatment. Flexible endoscopy is a prevalent choice for managing foreign objects, due to fewer complications. Rigid endoscopy is better suited for proximal and sharp objects. Barium esophagography has been suggested for suspected radiolucent ingested objects, but contrast studies pose a risk of aspiration and compromise subsequent endoscopy. Surgical removal is indicated in cases of perforation and especially after the failure of endoscopic removal, as was proven in our case [6-8].

## Conclusion

The diagnosis of chronic esophageal radiolucent foreign body ingestion is an extremely challenging entity in the pediatric population, in terms of diagnosis, differential diagnostic approach, and means of management. Specific questioning during medical history recording, regarding bizarre eating habits or certain psychosocial behavior is crucial in such cases, determining the extent of investigation and deciding and prioritizing the means of possible and feasible intervention.
